# Pomegranate Fruit Cracking during Maturation: From Waste to Valuable Fruits

**DOI:** 10.3390/foods12091908

**Published:** 2023-05-06

**Authors:** Caterina Fraschetti, Enkelejda Goci, Alexandru Nicolescu, Francesco Cairone, Simone Carradori, Antonello Filippi, Vincenzo Palmieri, Andrei Mocan, Stefania Cesa

**Affiliations:** 1 Department of Drug Chemistry and Technology, “Sapienza” University of Rome, P.le Aldo Moro 5, 00185 Rome, Italy; caterina.fraschetti@uniroma1.it (C.F.); francesco.cairone@uniroma1.it (F.C.); antonello.filippi@uniroma1.it (A.F.); stefania.cesa@uniroma1.it (S.C.); 2 Pharmacotherapeutic Research Center, Faculty of Medical Sciences, Aldent University, 1001 Tirana, Albania; enkelejda.goci@ual.edu.al; 3 Laboratory of Chromatography, Advanced Horticultural Research Institute of Transylvania, Faculty of Horticulture and Business for Rural Development, University of Agricultural Sciences and Veterinary Medicine, 400372 Cluj-Napoca, Romania; alexandru.nicolescu@usamvcluj.ro (A.N.); mocan.andrei@umfcluj.ro (A.M.); 4 Department of Pharmacy, “G. d’Annunzio” University of Chieti-Pescara, Via dei Vestini 31, 66100 Chieti, Italy; 5 Fratelli Palmieri Via Emanuele Filiberto, 56, Casalnuovo Monterotaro, 71033 Foggia, Italy; vincenzopalmieri658@gmail.com; 6 Department of Pharmaceutical Botany, “Iuliu Hațieganu” University of Medicine and Pharmacy, Gheorghe Marinescu Street 23, 400337 Cluj-Napoca, Romania

**Keywords:** pomegranate, punicalagin, ellagic acid, shelf-life, CIEL*a*b*, HS-SPME/GC-MS, α-glucosidase

## Abstract

The pomegranate is an emerging functional food which is nowadays becoming more and more commercially attractive. Each part of this fruit (peels, arils and seeds) has a specific phytocomplex, rich in anti-oxidant and anti-radical compounds. Among these, punicalagin and ellagic acid continue to be widely studied for their numerous beneficial effects on human health (anti-inflammatory effects, anti-diabetes activity, cardio-protection, cancer prevention). Despite their exceptionally valuable composition and high adaptability to different climatic conditions, pomegranate fruits are highly susceptible to splitting during different stages of ripening, so much so that an estimated 65% of the production may be lost. A “zero-kilometer” approach should therefore be adopted to utilize such a valuable product otherwise destined to be downgraded or even incinerated, with a very high environmental impact. The aim of this work is to highlight and compare the compositional differences between whole and split pomegranates belonging to the cultivar Dente di Cavallo, grown in Apulia (Italy), to assess a valuable role for this split fruit usually considered as waste. The arils and peels are subjected to extraction procedures and the extracts analyzed by CIEL*a*b*, HPLC-DAD and HS-SPME/GC-MS. Moreover, an assessment of the inhibitory activity against α-glucosidase, acetylcholinesterase and tyrosinase enzymes has also been applied. The data show a better chemical profile in split fruits (namely 60% more anthocyanin content than intact fruit) with very interesting results in terms of α-glucosidase inhibition. The juices obtained by squeezing are also compared to commercial juices (“Salus Melagrana” and “La Marianna”) processed from the same cultivar and subjected to the same protocol analysis.

## 1. Introduction

The nutritional potential of pomegranates as an emerging functional food is currently growing, together with consumer demand, making this fruit an interesting commercial area. In addition to the nutraceutical properties of its different botanical parts, *Punica granatum* represents a species that is highly adaptable to different climates, arid areas included. It is represented by many different genotypes, with domestic, wild or ornamental cultivars, sour or sweet characteristics and many other differences in terms of organoleptic parameters, such as peels, arils and juice color or fruit dimension [1,2]. Each part of this fruit (peels, arils and seeds) presents a highly specific composition when compared with other edible vegetables, generally presenting flavonoid content rich in anti-oxidant and anti-radical compounds. Pomegranate arils present an exceptionally high content of ellagitannins, mainly represented by punicalagins. Moreover, the peels are characterized by an ellagitannin content that is much higher than the arils (up to four or five times), shifted towards a more conspicuous ellagic acid content. Finally, the seeds represent an excellent source of conjugated isomers of linolenic acid, with punicic acid being the most represented. Punicalagins and ellagic acid continue to be largely studied and reviewed [3,4] for their numerous beneficial effects on human health (apoptosis induction in cancer cells, down-regulation of pro-inflammatory factors, anti-diabetes activity, cardioprotection, prevention of chronic diseases); similarly, conjugated isomers of linolenic acids and punicic acid [5,6] are under evaluation for their effects on lipid metabolism regulation and anti-obesity activity, in addition to anti-oxidant, anti-inflammatory, immunomodulatory, antiproliferative and anti-carcinogenic activities. Despite the exceptionally powerful phytocomplex component and the high adaptability of the shrubs to different climate conditions, pomegranate fruits are highly susceptible to cracking during different maturation stages, so much so that it is estimated that about 65% can be lost, depending on the growing conditions.

Some authors report in a recent review [7] that many different causes could be associated with this problem, such as environmental and nutritional factors, but it is also undoubtedly linked to irregular irrigation or water imbalance. High-temperature daytime and nighttime travel, especially in the final maturation step, can provoke excessive transpiration with consequent fruit splitting. Therefore, while consumer demand is growing, manufacturers face many obstacles in placing the fresh product on the market. If this problem could easily be bypassed using the split fruits for juice production, it is also true that these are more susceptible to attack by pathogens, with rapid alteration and degradation. Thus, a “zero-kilometer” approach should be adopted in order to use such a valuable product otherwise destined to be downgraded or even incinerated, with a very high environmental impact.

**Figure 1:**
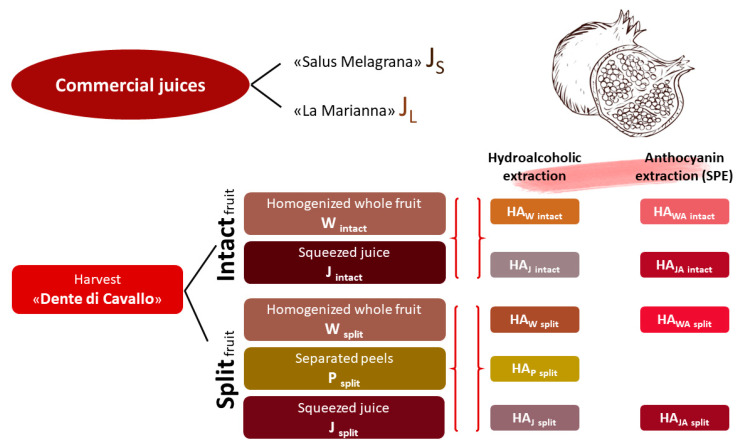
Flow chart.

In view of the impressive growth of its market, but also due to the underlying potentialities and problems linked to this crop, the present work aims to show the compositional differences between intact and split fruit in order to prevent the loss of the latter as waste [8]. Fruits belonging to “Dente di Cavallo” variety, the most prevalent cultivar in Italy, cultivated in the Apulia region, were directly harvested. The fruits were processed as such or separated into their constitutive parts (peels and arils), subjected to extraction procedures, analyzed in their polyphenolic composition, color and volatile and aromatic components. Finally, inhibitory activity against three important enzymes (tyrosinase, acetylcholinesterase and α-glucosidase) for therapeutic applications was also assessed through in vitro assays and compared with suitable reference compounds. In order to evaluate the quality of the obtained squeezed juice, the derived commercial juices “Salus Melagrana” and “La Marianna” were submitted to the same protocol analysis for comparison (Figure 1).

## 2. Materials and Methods

### 2.1. Materials

Ethanol, methanol and acetonitrile (HPLC-grade) were obtained from Merck Science Life s.r.l (Milan, Italy). All solvents and chemical standards used in this paper were analytical grade products purchased from Merck Science Life s.r.l (Milan, Italy) and were used without any further purification.

### 2.2. Samples

The pomegranate fruit (*Punica granatum* L.) cv. “Dente di cavallo”, harvested intact and split, and the pomegranate juice “Salus Melagrana” (Eurosalus Italia Srl—Via Francia 6G, Negrar, 37024 (Verona, Italy)) and “Succo La Marianna” were collected or obtained by cold pressing from Fratelli Palmieri, Casalnuovo M.ro (Foggia, Italy) in the Fortore River valley, a natural oasis for the protection of plant and animal biodiversity. The whole fruit (W), separated peels (P) and their squeezed juice (J), each obtained from five different pomegranates, were submitted to different analyses for the phytocomplex characterization. All the experiments were performed in quadruplicate. Both intact and split fruit were harvested to assess any differences in content or biological activity (Figure 1).

### 2.3. Hydroalcoholic Extraction

W and J, obtained both from the intact fruit and from the split fruit, and P, obtained from the split fruit, were submitted to the hydroalcoholic extraction procedure as previously described by Altieri et al. (2019) [9]. Samples (10 g) from approximately 10 kg were randomly selected from different bulks representative of the whole seasonal harvest, blended and extracted with 40 mL of ethanol:acidified water (5% acetic acid) in 3:1 (*v*:*v*) ratio, with stirring for 1 h at room temperature in the dark. The extraction mixture was decanted, filtered and evaporated at 40 °C under vacuum and stored at 4 °C until the analyses were performed (samples of HA_W_, HA_P_ and HA_J_).

### 2.4. Anthocyanin Extraction

HA_W_, HA_P_ and HA_J_ were subjected to solid phase extraction (SPE) for the purification and quantification of anthocyanin. The extraction was performed using a Discovery^®^ DSC-18 SPE Tube column (Merck Life Science, S.r.l., Milan, Italy), according to Yılmaz et al. (2015) [10], with substantial modifications. The column was conditioned beforehand with 5 mL of ethyl acetate, 5 mL of methanol (5% CH_3_COOH *v*/*v*) and, finally, with 2 mL of H_2_O (5% CH_3_COOH *v*/*v*). Then, about 100 mg/mL of the samples was loaded into the column. The column was washed with 6 mL of H_2_O (5% CH_3_COOH *v*/*v*) and 12 mL of ethyl acetate, which were subsequently discarded. Finally, the anthocyanin fraction was eluted with 4 mL of methanol (5% CH_3_COOH *v*/*v*). The obtained fractions were concentrated under reduced pressure at a controlled temperature of 40 °C, weighed and stored at 4 °C until HPLC-DAD analyses were performed (split and intact samples of HA_WA_ and HA_JA_).

### 2.5. Colorimetric Analysis and Accelerated Test of Food Shelf-Life

W and J, HA_w_, HA_P_ and HA_J_, and the commercial juices “Salus Melagrana” (J_S_) and “Succo La Marianna” (J_L_) were submitted to colorimetric CIEL*a*b* analysis with a colorimeter X-Rite MetaVue^TM®^ equipped with a full-spectrum LED illuminant and an observer angle of 45°/0° imaging spectrophotometer. The analyses were conducted according to Recinella et al. (2021) [11]. The analyses of the juices J_S_ and J_L_ were performed at the time of delivery (t°) and weekly for five weeks, keeping the samples in the darkness at 37 ± 2 °C.

### 2.6. HPLC-DAD Analysis

The dried extracts taken from the intact and split samples HA_w_, HA_P_ and HA_J_ and the SPE extracts HA_WA_ and HA_JA_ were weighed and dissolved in a known volume of hydroalcoholic solution (5 mg/mL). The resulting solutions and the commercial juices, such as J_S_ and J_L_, were filtered with a Millex^®^—LG filter (Low Protein Binding Hydrophilic PTFE 0.20 µM Membrane) (Merck Science Life, S.r.l, Milan, Italy), injected and analyzed with an HPLC-DAD (Perkin Elmer, Milan, Italy), equipped with an LC Series 200 pump, a Series 200 DAD, and a Series 200 autosampler, including Perkin Elmer TotalChrom software for data tracking. Analyses were performed on HA_w_, HA_P_, HA_J_, J_S_ and J_L_ at 280 nm for the identification of gallic acid and at 360 nm for the identification of the ellagitannin profile. HA_WA_ and HA_JA_ were analyzed at 520 nm for the identification of anthocyanins. A Luna RP-18, 3 µm column was used, with a linear gradient consisting of acetonitrile and acidified water (5% formic acid), from 100% to 15% aqueous phase in 60 min, at a flow rate of 1.0 mL/min. Calibration curves were expressed in µg/mL and were constructed for gallic acid (y = 15.51x + 37.06; R^2^ 0.9987), punicalagin (α + β anomers) (y = 3.83x − 49.95; R^2^ 0.9998), ellagic acid (y = 16.86x + 1.22; R^2^ 0.9994) and cyanidin-3-*O*-rutinoside (y = 16.58x + 34.53; R^2^ 0.9987). Extraction yields of anthocyanins, though quantified on SPE extracts, were finally expressed in relation to the hydroalcoholic extracts to compare the differently obtained data.

### 2.7. HS-SPME/GC-MS Analysis

Dried HA_w_ and HA_J_ samples (0.3 mg), taken from both the split and intact fruit, and HA_P split_ were introduced in 4 mL vials and allowed to equilibrate for 20 min in a thermostat bath set at 80 °C. The equilibration step was followed by the exposure of the CAR-DVB-PDMS fiber to the headspace of the vial for 20 min at 80 °C. Finally, the analytes were allowed to desorb from the fiber exposed into the inlet of an Agilent Technologies 6850 gas chromatograph, coupled with an Agilent Technologies 5975 mass spectrometer, for 0.5 min. The following gas chromatographic layout was used: column, HP-5MS (30 m × 0.25 mm inner diameter, film thickness 0.25 µm); inlet temperature, 260 °C; injection mode, splitless (the split vent was opened after 0.5 min and the split ratio set at the 20/1 value); flow rate of the helium carrier gas (99.995% purity), 1.0 mL/min; oven temperature starting from 40 °C, after 5 min raised to 200 °C at 5 °C/min, and kept at this final temperature for 60 min. Mass spectrometry parameters were set as follows: EI energy, 70 eV; source temperature, 230 °C; quadrupole temperature, 150 °C; the mass scan was carried out over the 50–350 *m*/*z* range.

The two-level identification of the eluted compounds started from comparing the experimental EI spectra with those collected in both commercial (FFNSC 3) and free databases (NIST 11, Flavor2). The Kovats index (KI) was used as a second parameter to confirm the MS-based identification of the analytes. KIs were measured using a mixture of *n*-alkanes (C7–C40) in the same chromatographic set-up, and then compared with values reported in the FFNSC 3 and NIST 11 databases. Chromatographic peaks with a S/N ratio above 3 were manually integrated without any further modification.

### 2.8. Enzyme Inhibitory Activity

The samples were subjected to enzyme inhibitory assays against three fundamental enzymes with implications in human pathologies: α-glucosidase, acetylcholinesterase and tyrosinase, using in vitro assays. All the results were expressed in terms of IC_50_ (μg/mL), considering a dilution in the microplate, and not the original vial dilution. The percentages of inhibition (*I*, expressed as %) for every enzyme inhibition assay were calculated using the formula below:

$$I\left(\%\right)=\frac{A_{control}-A_{sample}}{A_{control}}\times 100$$

where *A_control_* is the absorbance of the control solution and *A_sample_* is the absorbance of the sample, against blanks. Inhibitory activity against α-glucosidase was assessed using a previously described protocol. Then, 50 μL of different concentrations of the same extract were mixed with 50 μL of α-glucosidase (in a pH 6.8 phosphate buffer solution, PBS). After adding 50 μL of the substrate (4-nitrophenyl-β-D-glucopyranoside PNPG, 10 mM in PBS), the reaction mix was incubated for 5 min at 37 °C, and the absorbance was read at 405 nm. The same protocol was applied for acarbose as positive control [12].

For acetylcholinesterase, a protocol based on Ellman’s method was used, in which 25 μL of diluted sample was mixed with 50 μL of Tris-HCl buffer (pH 8.0) and 125 μL of 5,5-dithio-bis-(2-nitrobenzoic acid) (DTNB, 0.9 mM). Next, 25 μL of the enzyme was added, and the reaction mixture was incubated for 15 min at 25 °C. After the first incubation, the samples were mixed with 25 μL of acetylthiocholine iodide (ATCI, 4.5 mM) and then re-incubated for 10 min at 25 °C. The absorbance was read at 405 nm. The same protocol was applied for galantamine as positive control [12].

For the inhibition of tyrosinase, 40 μL of different concentrations from the same extract were mixed with 80 μL of PBS (with pH 6.5) and with 40 μL of enzyme in PBS, followed by an incubation for 10 min at 25 °C. After the incubation time, 40 μL of L-DOPA (10 mM, in PBS) was added to the mixture, and another incubation for 20 min at 25 °C was applied. The absorbance was read at 475 nm. The same protocol was applied for kojic acid as positive control [12].

## 3. Results and Discussion

### 3.1. Polyphenols Extraction

Split and intact whole fruits and separated peels were homogenized (W and P) or squeezed (J). The resulting homogenates and juices were submitted to a mild extraction method as previously reported in the Materials and Methods section (HA_W_, HA_P_ and HA_J_). The hydroalcoholic extraction yield ranged from 10% to 12% by dry weight in HA_W_ and HA_P_ accounting for the sugar content and not directly correlated to the polyphenolic content, as detailed below by HPLC analyses. The extraction yields afforded by starting from HA_J_ showed higher ranges, between 15–16%, accounting for the more concentrated sugar content and soluble fibers of the edible part with respect to the peels represented in the whole fruits. No significant differences were shown among extracts from split or intact fruits and, on the whole, data are comparable with our previously obtained results on different pomegranate cultivars [9,13].

Considering the extraction yields related to the solid phase extraction (SPE) of anthocyanins by the hydroalcoholic extracts, yields ranging from 1.1 to 2.8% were obtained. In any case, these are only indicative because, as the anthocyanins were concentrated and made perceptible using HPLC-DAD analysis, other polyphenols and flavonoids were still present. The highest yields of SPE extracts were shown in samples obtained from split fruits (HA_WA split_, 2.8%; HA_JA split_, 1.7%) and effectively correlated with the higher anthocyanin amount found using HPLC-DAD analysis.

### 3.2. Colorimetric Analysis

As is well known, different pigments deeply characterize pomegranate fruit components. Anthocyanins, contained in arils, confer a brilliant red color, and yellow-brown ellagitannins are represented in both the arils and peels, contributing to or determining their color [14].

The W _intact_, W _split_, J _intact_, J _split_, HA_W intact_, HA_W split_, HA_J intact_, HA_J split_, HA_P split_, J_S_ and J_L_ samples were submitted to colorimetric CIEL*a*b* analysis. The J_S_ and J_L_ juices were further submitted to a shelf-life study, and the color differences were monitored over time. The CIEL*a*b* parameters are reported in Table 1.

With regard to the homogenized samples (W _intact_ and W _split_) and juices (J _intact_, J _split_, J_S_ and J_L_), the L* parameter varies between 12.41 and 44.58, a* between 11.00 and 38.26 and b* between 15.29 and 31.81.

**Figure 2:**
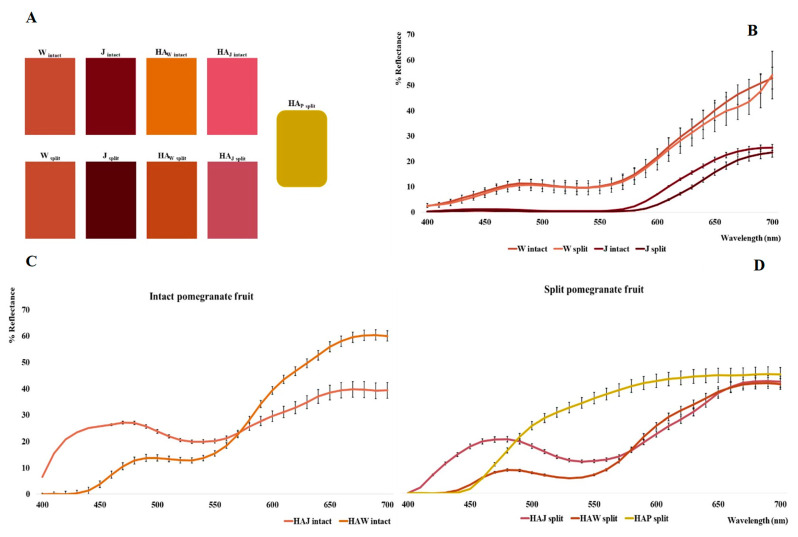
Palette of analyzed samples (**A**); reflectance curves related to W and J (intact and split) samples (**B**); reflectance curves related to obtained hydroalcoholic extracts (**C**,**D**).

Specifically, there is no statistically significant difference (Figure 2B) between samples W _intact_ and W _split_ (ΔE = 5.32), and only a slight difference is observed between J _intact_ and J _split_ (ΔE = 7.86). This mainly concerns the range between 600 and 650 nm, where J _split_ shows a lower reflectance curve, probably due to a higher concentration of anthocyanin pigments. In fact, while the CIEL*a*b* parameters are similar in the W series (L*, 44.58 vs. 43.63; a*, 21.20 vs. 20.99; b* 17.90 vs. 19.06), in the J series, in addition to an increase in L* (12.41 rises to 19.51), a significantly higher value of a* is also observed (34.35 rises to 38.26).

In the hydroalcoholic samples (HA_W intact_, HA_W split_, HA_J intact_, HA_J split_, HA_P split_), L* values ranged between 41.39 (HA_W split_) and 63.96 (HA_P split_), showing the highest luminance values compared to W and J before the extraction step. In this regard, as indicated from the color palette shown in Figure 2A, it is possible to observe a brighter and more brick red color for HA_W_ samples than for W samples, with a greater difference for HA_W intact_ (ΔE = 31.65) with respect to HA_W split_ (ΔE = 21.36). Conversely, HA_J_ samples present a lighter and more opaque reddish color than the intense mahogany coloration of the J samples, being more pronounced in HA_J intact_ (ΔE = 53.14) with respect to HA_J split_ (ΔE = 43.09). Completely different coloration is obviously presented by the HA_P_ sample from the peels, which tends towards dark yellow.

In addition, the a* parameter drops to −1.66 in HA_P split_ and rises to 28.86 in HA_W split_, whereas the b* parameter varies deeply between 0.29 (HA_J intact_) and 62.59 (HA_P split_). Tendentially, the a* and b* parameters are always higher in the split series, with the exception of HA_W split_ (32.11 vs. 48.14). This difference could be related to a higher concentration of pigments (both anthocyanins and ellagitannins) in split samples than in intact ones. It is also observed that the a*/b* ratios in the HA_J_ series are higher than in the HA_W_ and HA_P_ series, in relation to a higher anthocyanin content with respect to ellagitannins (see also the HPLC-DAD analysis). Conversely, the higher yellow positive b* parameter could be associated with ellagitannins [15].

The highest value of b* is found in HA_P split_, richer in ellagitannins as further demonstrated by the HPLC-DAD analysis. This also correlates with reflectance curves shown by Figure 2C,D. Indeed, the curves related to the hydroalcoholic extracts of the split pomegranates compared with the intact samples are lower in the region around 650 nm. In particular, there is a marked 19% decrease in reflectance for HA_W split_, and a slight decrease (about 2%) for HA_J split_, confirming the pigment’s prevalence in the split samples; this behavior is evident in the redder juice samples obtained by squeezing, due to the high anthocyanin content represented in pomegranate arils [16,17]. The present data are partially comparable with those reported in the literature [13].

**Figure 3:**
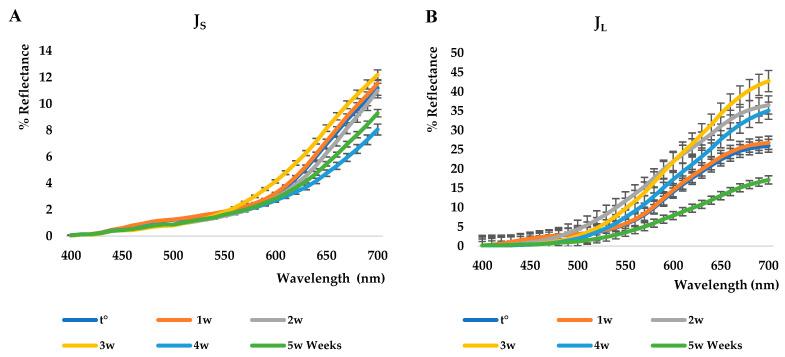
Shelf-life study on Salus Melagrana (J_S_) (**A**) and La Marianna (J_L_) juices (**B**).

Figure 3A,B shows the reflectance curves related to the shelf-life study conducted on J_S_ and J_L_ juices kept at 40 °C for five weeks. Regarding J_S_, a color change is observed after the first week, and it remains constant until the third week, when a bleaching phenomenon is observed. On the contrary, around the fourth and fifth weeks, a darkening is observed, probably associated with a higher concentration of ellagitannins (see also HPLC data). A substantially different trend is registered for J_L_. In fact, darkening is found in the first week, followed by bleaching until the third week, and then darkening again around the fifth week.

### 3.3. HPLC-DAD Analysis

**Figure 4:**
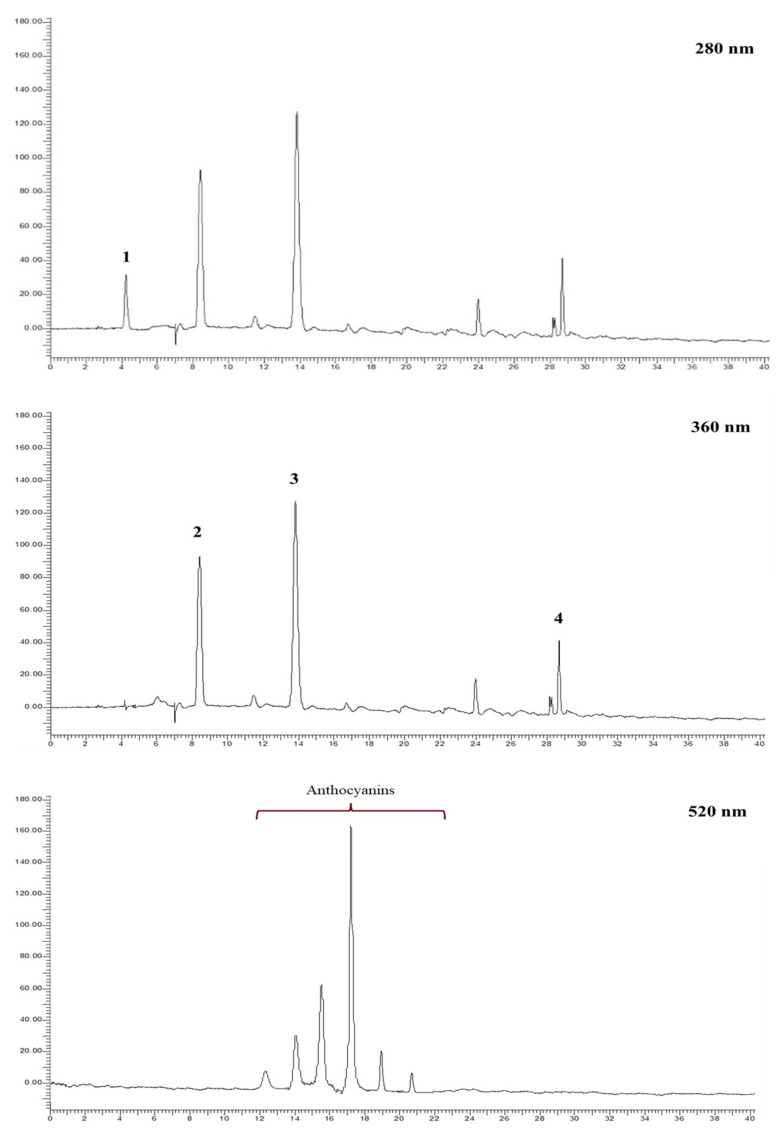
Example of HA_W split_ chromatograms at 280 nm (1. Gallic acid) and at 360 nm (2. α-punicalagin, 3. β-punicalagin, 4. ellagic acid), and HA_WA split_ chromatograms at 520 nm for the identification of anthocyanins.

The different hydroalcoholic extracts obtained from pomegranate fruits of “Dente di Cavallo” and the two related commercial juices were subjected to HPLC-DAD analysis. The analyses were performed at 280 nm for the identification of phenolic acids, at 360 nm for the identification of the ellagitannin profile, mainly represented by punicalagin (α + β) and ellagic acid, and at 520 nm for the identification of the anthocyanins. Compounds were identified by external standard or by comparison with the literature [18,19]. As anthocyanins were not directly detectable from these extracts, they were analyzed after a further step of solid phase extraction, which made it possible to concentrate and quantify these pivotal compounds as well. Examples of chromatograms related to the HA_W split_ and HA_WA split_ samples are shown in Figure 4.

The quantification of ellagitannins, reported as mg/g dry extract (Table 2), evidenced relevant differences in the range of 3–17 mg/g dry extract by juices, 21–32 mg/g dry extract by whole fruits, up to the maximum amount (77 mg/g dry extract) in the peel. Very low values were found for juices, with the exception of J_S_ (17 mg/g by dry extract). In fact, significant differences could be seen among the three applied work-up methodologies. Juices obtained by simple pressing of the fruit (HA_J_, both from intact and split fruits, and J_L_) show very low values of punicalagin and only in the case of J_L_ a very slight amount of ellagic acid (<0.1 mg/g dry extract). On the contrary, in J_S_ obtained by compression of the whole fruit, values of punicalagin and ellagic acid (17 and 1 mg/g dry extract, respectively) comparable to those recorded in the whole fruit were shown. The anthocyanin amount, yielded in relation to hydroalcoholic extracts, as well as quantified in SPE extracts, on the other hand, varied among 1 and 37 µg/g dry HA extract, reaching maxima in juice-related extracts. Anthocyanins were not detected in the J_S_ and J_L_ samples. The reported data agree with our previous work [9].

**Figure 5:**
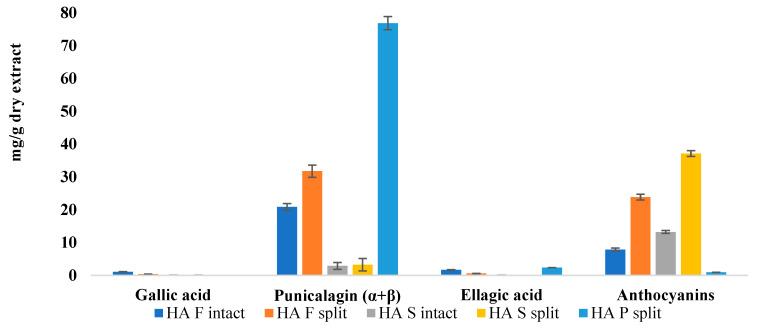
Effect of harvesting date on pomegranate phytocomplex.

Further considerations were made by analyzing extracts from the intact or split fruit. As shown in Figure 5, the amount of ellagitannins, especially punicalagins, and anthocyanins appears to be higher in extracts from split fruits with respect to intact fruits. This information is very interesting, as it is related to the fact that ripening and storage can influence the matrix phytocomplex [20,21]. Such evidence could confirm what was observed in the colorimetry, as lower reflectance curves of split samples correspond to higher concentrations of ellagitannins and anthocyanins.

**Figure 6:**
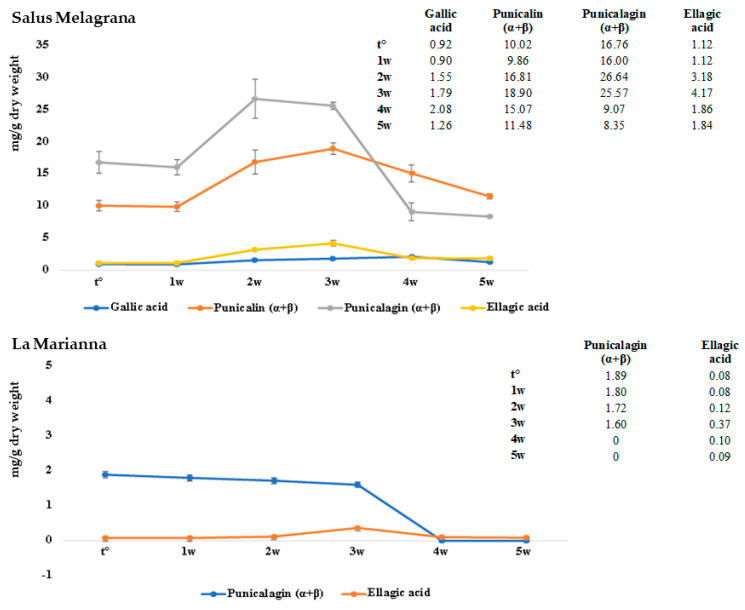
Shelf life of “Salus Melagrana” and “La Marianna” juices.

The shelf-life study was conducted by storing the samples at 37 °C for 5 weeks and evaluated using both colorimetric and HPLC-DAD analyses. As shown in Figure 6, in both J_S_ and J_L_, a slight increase in ellagic acid was observed in the first two weeks, and a decrease was observed until the stabilization observed at around five weeks.

With regard to punicalagin, whereas in J_L_ there is a sharp drop after three weeks, in J_S_ there is a slight decrease in the first weeks and then it stabilizes around four to five weeks at lower values with respect to the t°. At the same time, punicalin, identified only in J_S_, increases as punicalagin decreases between the second and third week, coming back to the initial values around five weeks. In conclusion, a decrease in punicalagin could be observed, whereas punicalin and ellagic acid tend to reestablish at the initial values, in an observation period of five weeks at 37 °C. Gallic acid, represented in smaller quantities comparable to those of ellagic acid, appears quite stable for the duration of the whole experiment.

### 3.4. HS-SPME/GC-MS Analysis

**Figure 7:**
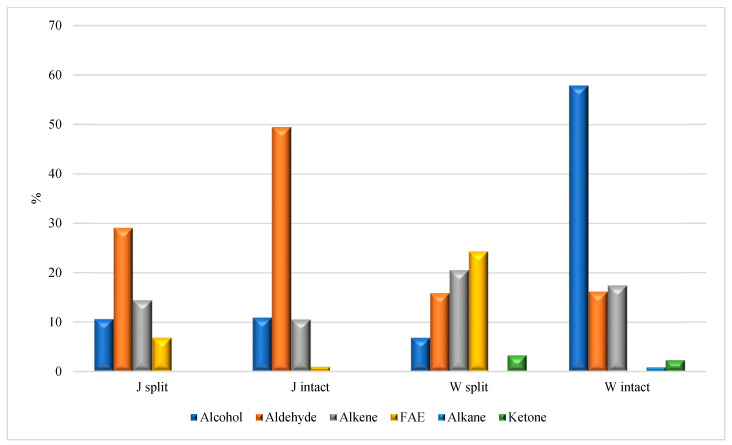
Comparison in the distribution of the prevailing classes of compounds in split and intact HA_J_ and HA_W_.

**Figure 8:**
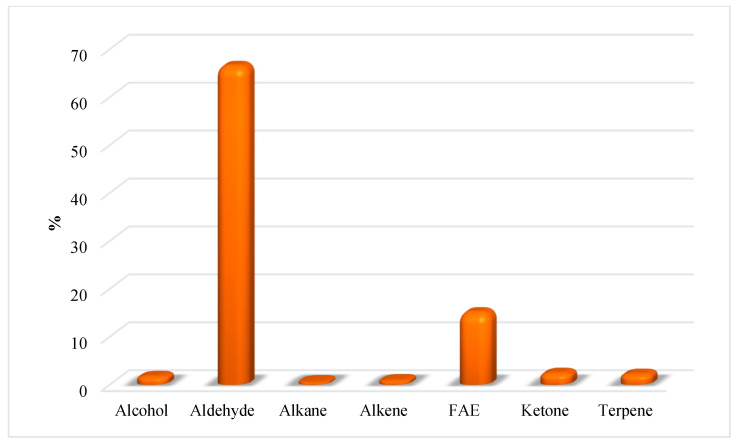
Distribution of the prevailing classes of compounds in HA_P split_.

GC-MS is the technique of choice to detect and identify apolar and medium-polarity metabolites arising from vegetable and food matrices [22,23]. In the present study, the HS-SPME/GC-MS analysis of split and intact HA_J_ and HA_W_ samples made it possible to identify several compounds clustered according to their chemical classification (Table 3 and Table 4), and these are reported in Figure 7. Aldehydes represent the prevalent class of chemicals in the HA_J_ samples (Figure 7), with a larger abundance in the HA_J intact_, mainly due to the presence of hexanal (not detected in HA_J split_), nonanal (6.4 vs. 11.2% in HA_J split_ and HA_J intact_, respectively) and decanal (7.2 vs. 12.4% in HA_J split_ and HA_J intact_, respectively). The HA_W_ samples differ mainly due to the abundance of alcohol (6.8 vs. 57.9% in HA_W split_ and HA_W intact_, respectively), which is completely ascribable to the presence of carvacrol in HA_W intact_, and the FAE distribution (24.3% in HA_W split_ but totally absent in HA_W intact_). The absence of carvacrol, even in trace amounts, in all the other analyzed samples is a reasonable clue of its presence in the seeds of the pomegranate. A further comparison between the four analyzed samples reveals the following details: (i) the alkene distribution in the four analyzed samples ranges between 10.5 in HA_J intact_ and 20.5 in HA_W split_; (ii) the FAE class was detected in the HA_J split_ and HA_W split_ samples (6.8 and 24.3%, respectively), but was poorly concentrated (0.9%) and absent in HA_J intact_ and HA_W intact_, respectively; (iii) methoxy phenyl oxime, a compound naturally occurring in food matrices but also recognized as a SPME fiber contaminant, was detected in all samples except for HA_W intact_ [24,25]. Lastly, the HS-SPME/GC-MS analysis of the HA_P split_ (Table 5 and Figure 8) pointed to aldehydes as the most abundant class of compounds (66.4%), with nonanal and decanal comprising the largest part (47.3%).

### 3.5. Enzyme Inhibitory Activity

The HA_W intact_, HA_W split_, HA_J intact_, HA_J split_ and HA_P split_ samples were submitted to enzymatic inhibitory activity assays, in an attempt to assess the potential to inhibit three important enzymes with implications in human physiopathology: α-glucosidase, acetylcholinesterase and tyrosinase (Table 6).

**Figure 9:**
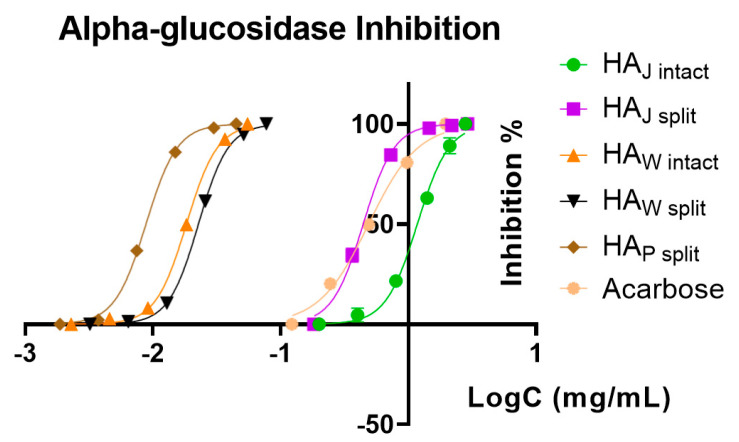
Logarithmic inhibitory curves for the pomegranate samples, as well as for acarbose as positive control.

As a general trend, the HA_J intact_ and HA_J split_ samples showed the lowest inhibitory activity among all, and for acetylcholinesterase and tyrosinase, we could not determine any activity. In all the samples, the inhibitory activity against acetylcholinesterase and tyrosinase was weak, with values at least 100 times higher than the positive controls used. For the α-glucosidase enzyme, the only sample with activity lower than the positive control (acarbose) was HA_J intact_, with an IC_50_ of 294.25 μg/mL. Regarding the same enzyme, the sample HA_P split_ showed the highest inhibitory activity, with an IC_50_ of 2.20 μg/mL, followed by HA_W intact_ and HA_W split_. These results can also be observed in Figure 9, where the logarithmic inhibition curves show better activity than acarbose (IC_50_ of 122.27 μg/mL) for all samples, with the exception of HA_J intact_.

Compounds with inhibitory activity against α-glucosidase, such as acarbose, voglibose and miglitol, have the potential to be used therapeutically in delaying glucose absorption (postprandial glycemia) from the gastrointestinal tract as adjunctive therapy of type 2 diabetes mellitus. This enzyme digests starches and carbohydrates, lowering insulin demand and sustaining a long-term release of GLP-1. Commercially available competitive and reversible inhibitors can limit the progression of diabetes but do not have any effects on pre-existing cardiovascular disease.

Kam et al. (2013) [26] studied the α-glucosidase inhibitory activity of different parts of the pomegranate, showing that some phenolic species, including ellagic acid, can selectively inhibit this enzyme. Interestingly, it was also identified that the highest amount of ellagic acid and punicalagin in the HA_P split_ sample corresponded with the highest inhibitory activity. Thus, the findings of this study highlight that the chemical composition of the phenolic content is a factor influencing the selective inhibitory effect against α-glucosidase. Furthermore, Çam and İçyer (2015) [27] found that phenolic species of pomegranate peels had an IC_50_ of 5.56 μg/mL for α-glucosidase, which are in line with our results. Other phenolic derivatives display inhibitory activity against this enzyme, for example, ellagitannins, ellagic acid and punicalagin from the peels [28,29,30]. The applicability of pomegranate peels as a by-product can also be further enhanced with a suitable formulation, for example, by microencapsulation [31].

## 4. Conclusions

This work allowed for a better valorization of the composition and functionality of the selected pomegranate cultivar “Dente di Cavallo”, widely consumed for its excellent nutritional properties. The whole fruit, separated peels and juice produced by homemade pressing of intact or split fruits, as well as two commercial juices, were analyzed. The two commercial juices, obtained through substantially different procedures, showed significant phytocomplex differences. The shelf-life study, conducted on color change, also demonstrated that the sample browning was directly related to the increase in ellagitannins. In intact fruits, a greater number of different volatile molecules were identified, for example, aldehydes (nonanal, decanal) in juices and peels and carvacrol in the whole fruit. Interestingly, an unneglectable amount of FAE was detected exclusively in the HA_W split_.

Collectively, data showed a richer chemical profile for extracts obtained from split fruit, both in terms of ellagitannins and anthocyanins. This higher bio-compound profile, especially for pomegranate juice, also leads to good health-promoting activity, most evident in α-glucosidase inhibition (IC_50_: HA_J split_ vs. HA_J intact_, 110.92 vs. 294.25). Data confer an added value to this underutilized or even discarded product, suggesting that the adoption of a “zero-kilometer” approach could be carefully considered, thereby preventing its disposal and rapid deterioration and yielding a high valuable product. Finally, concerning functionality, the extracts obtained from peels, much richer in ellagitannins, showed excellent inhibitory properties against the α-glucosidase enzyme. These results, better than those exerted by acarbose, both for intact and split derived products, suggest their useful application in type 2 diabetes prevention and in the reduction in post-prandial glucose concentrations, extending their nutritional value (conventional functional foods). With respect to the current arsenal against this enzyme, the pomegranate phytocomplex contained in the juices could help avoid various side effects (diarrhea, abdominal discomfort, flatulence and bloating). Split fruits preserve this bioactivity, thus proposing themselves as valuable waste which needs to be further explored for its positive impact on an individual’s health.

## Figures and Tables

**Table 1:** Colorimetric data of the analyzed samples.

	L*	a*	b*	C_ab_*	h_ab_
W _intact_	44.58 ± 2.90	21.20 ± 0.11	17.90 ± 0.18	27.66 ± 0.85	40.53 ± 1.23
W _split_	43.63 ± 4.51	20.99 ± 0.86	19.06 ± 1.88	26.56 ± 0.85	40.23 ± 1.05
J _intact_	12.41 ± 0.11	34.85 ± 2.17	15.29 ± 2.24	39.24 ± 1.06	22.81 ± 1.03
J _split_	19.51 ± 0.49	38.26 ± 0.57	17.84 ± 1.34	40.14 ± 1.23	24.11 ± 2.07
HA_W intact_	53.36 ± 2.66	25.98 ± 1.29	48.14 ± 2.41	54.73 ± 2.74	61.85 ± 3.09
HA_W split_	41.39 ± 2.07	28.86 ± 1.44	32.11 ± 1.61	43.78 ± 2.19	48.58 ± 2.43
HA_P split_	63.96 ± 3.19	−1.66 ± 0.08	62.59 ± 3.12	62.62 ± 1.87	91.52 ± 2.74
HA_J intact_	55.61 ± 2.78	7.79 ± 0.39	0.29 ± 0.01	7.93 ± 0.39	12.93 ± 0.65
HA_J split_	47.29 ± 2.36	14.83 ± 0.74	1.79 ± 0.08	16.15 ± 0.81	31.20 ± 1.56
J_S_	16.40 ± 0.49	11.00 ± 0.33	18.26 ± 0.55	21.32 ± 0.64	58.93 ± 1.76
J_L_	33.25 ± 0.99	22.75 ± 0.68	31.81 ± 0.95	39.11 ± 1.17	54.42 ± 1.63

**Table 2:** Qualitative–quantitative analysis of the obtained pomegranate extracts, expressed in mg/g dry extract.

	Gallic Acid	Punicalin (α + β) *	Punicalagin (α + β)	Ellagic Acid	Anthocyanins **
HA_W intact_	1.10 ± 0.05	NI	20.83 ± 1.04	1.71 ± 0.09	7.87 ± 0.44
HA_W split_	0.41 ± 0.03	NI	31.75 ± 1.89	0.58 ± 0.002	23.87 ± 3.11
HA_P split_	NI	NI	76.84 ± 3.84	2.36 ± 0.10	0.95 ± 0.01
HA_J intact_	0.01 ± 0.001	NI	3.24 ± 0.02	NI	13.25 ± 0.87
HA_J split_	0.01 ± 0.001	NI	2.91± 0.03	NI	37.13 ± 4.14
J_S_	0.92 ± 0.07	10.02 ± 0.83	16.76 ± 1.70	1.12 ± 0.07	NI
J_L_	NI	NI	1.89 ± 0.05	0.08 ± 0.007	NI

NI: Not Identified; * expressed as punicalagin equivalents; ** expressed as µg/g dry extract of cyanidin-3-rutinoside.

**Table 3:** HS-SPME/GC-MS analysis of pomegranate juice (split and intact fruit).

Compound	Class	Area %		RI		RI_L_ ^a^
		Split	Intact	Split	Intact	
Hexanal ^b^	Aldehyde	-	7.5	-	-	-
Furfural ^b^	Aldehyde	12.9	15.2	-	-	-
Methoxy phenyl oxime ^b^	Other	17.7	17.1	935	939	-
Octanal	Aldehyde	2.6	2.5	1013	1013	1006
2-Ethyl hexanol	Alcohol	-	1.6	-	1044	1030
Nonanal	Aldehyde	6.4	11.2	1113	1113	1107
Dodecene	Alkene	2.6	1.2	1196	1196	1191
Decanal	Aldehyde	7.2	12.4	1214	1214	1208
Tetradecene	Alkene	7.0	4.5	1396	1396	1392
Dodecanal	Aldehyde	-	0.7	-	1418	1410
Diisopropyladipate	FAE	4.1	-	1465	-	1464
2,4-bis(1,1-dimethylethyl)-phenol	Alcohol	3.1	2.3	1528	1529	1521
Tridecanol	Alcohol	7.5	7.0	1597	1597	1580
Octyl octanoate	FAE	2.7	0.9	1787	1788	1781
Octadecene	Alkene	4.8	3.8	1798	1798	1793
Diisobutyl phthalate	Other	9.4	2.1	1885	1888	1868
Eicosene	Alkene	-	1.0	-	1999	2000
Unknown		12.0	8.5			
**Class**						
Alcohol		10.6	10.9			
Aldehyde		29.1	49.5			
Alkene		14.4	10.5			
FAE		6.8	0.9			

^a^ RI, reported in the literature; ^b^ MS as the only identification method.

**Table 4:** HS-SPME/GC-MS analysis of pomegranate whole fruit (split and intact fruit).

Compound	Class	Area %		RI		RI_L_ ^a^
		Split	Intact	Split	Intact	
Methoxy phenyl oxime ^b^	Other	7.5	-	940	-	-
6-Methyl-hept-5-en-2-one	Ketone	3.3	-	986	-	975
Octanal	Aldehyde	3.7	-	1013	-	1006
2-Ethyl-hexanol	Alcohol	-	8.0	-	1044	1030
3,4-Dimethyl-2-cyclohexen-1-one ^b^	Ketone	-	2.3	-	1091	-
Nonanal	Aldehyde	11.6	7.4	1112	1113	1107
Dodecene	Alkene	2.0	5.1	1196	1196	1191
Dodecane	Alkane	-	0.9	-	1204	1200
Decanal	Aldehyde	15.9	6.1	1213	1214	1208
Carvacrol	Alcohol	-	43.2	1320	-	1317
Tetradecene	Alkene	17.9	12.3	1396	1396	1392
Diisopropyladipate	FAE	2.4	-	1465	-	1464
Dodecanal	Aldehyde	-	2.7	-	1418	1410
Dodecanol	Alcohol	1.3	-	1486	-	1476
Tridecanol	Alcohol	5.5	6.7	1597	1597	1580
Octyl octanoate	FAE	21.9	-	1787	-	1781
Octadecene	Alkene	0.6	-	1798	-	1793
Diisobutyl phthalate	Other	1.6	-	1885	-	1868
Unknown		4.9	5.3			
**Class**						
Alcohol		6.8	57.9			
Aldehyde		15.9	16.2			
Alkane		-	0.9			
Alkene		20.5	17.4			
FAE		24.3	-			
Ketone		3.3	2.3			

^a^ RI, reported in the literature; ^b^ MS as the only identification method.

**Table 5:** HS-SPME/GC-MS analysis of pomegranate peel (split fruit).

Compound	Class	Area %	RI	RI_L_ ^a^
Heptanal	Aldehyde	2.2	913	906
Methoxy phenyl oxime ^b^	Other	2.7	940	-
1-Chloro-heptane	Other	0.8	965	962
Benzaldehyde	Aldehyde	2.8	976	960
6-Methyl-hept-5-en-2-one	Ketone	1.1	1000	986
Octanal	Aldehyde	10.3	1012	1006
α-terpinene	Terpene	0.4	1022	1018
*p*-Cymene	Terpene	1.1	1031	1025
5-Methyldecane	Alkane	0.5	1062	1058
γ-Terpinene	Terpene	0.9	1065	1058
1-Chloro-octane	Other	1.4	1067	1066
Octanol	Alcohol	0.6	1086	1076
3,4-Dimethyl-cyclohexen-1-one	Ketone	1.2	1090	1100
Nonanal	Aldehyde	23.5	1112	1107
1-Chlorononane	Other	1.1	1169	1154
Dodecene	Alkene	0.8	1196	1191
Decanal	Aldehyde	23.8	1213	1208
2E-Decenal	Aldehyde	1.6	1272	1265
Undecanal	Aldehyde	0.7	1286	1286
8Z-Undecenal	Aldehyde	1.5	1374	1365
Trans-α-bergamotene	Terpene	0.6	1445	1432
Diisopropyladipate	FAE	1.9	1466	1464
Dodecanol	Alcohol	0.4	1486	1476
2,4-Bis(1,1-dimethylethyl)-phenol	Alcohol	0.3	1529	1521
Tridecanol	Alcohol	0.3	1597	1580
Diisopropylphthalate	Other	0.3	1614	1633
Octyl octanoate	FAE	13.1	1787	1781
Diisobutylphthalate	Other	0.4	1885	1868
Unknown		3.6		
**Class**				
Alcohol		1.6		
Aldehyde		66.4		
Alkane		0.5		
Alkene		0.8		
FAE		15.0		
Ketone		2.3		
Terpene		2.1		

^a^ RI, reported in the literature; ^b^ MS as the only identification method.

**Table 6:** Results of the enzymatic inhibitory activity (expressed as IC_50_ in μg/mL).

	α-Glucosidase	Acetylcholinesterase	Tyrosinase
**HA_W intact_**	4.58	259.8	609.2
**HA_W split_**	5.68	379.2	722.2
**HA_P split_**	2.20	309.8	416.2
**HA_J intact_**	294.25	NI	NI
**HA_J split_**	110.92	NI	NI
**Positive control**	Acarbose: 122.27	Galantamine: 0.000185	Kojic acid: 4.44

NI = no inhibition (less than 50% inhibition at 1000 μg/mL).

## Data Availability

Data is contained within the article.
